# Postpartum depression and health-related quality of life: a Saudi Arabian perspective

**DOI:** 10.7717/peerj.14240

**Published:** 2022-10-14

**Authors:** Mansour Almuqbil, Nora Kraidiye, Hatoun Alshmaimri, Amerah Ali kaabi, Atheer Almutiri, Abeer Alanazi, Ayat Hjeij, Abdulhakeem S. Alamri, Wala F. Alsanie, Majid Alhomrani, Syed Mohammed Basheeruddin Asdaq

**Affiliations:** 1 Department of Clinical Pharmacy, College of Pharmacy, King Saud University, Riyadh, Riyadh, Saudi Arabia; 2 AlMaarefa University, Dariyah, Saudi Arabia; 3 Department of Clinical Laboratory Sciences, The Faculty of Applied Medical Sciences, Taif University, Taif, Saudi Arabia; 4 Centre of Biomedical Sciences Research (CBSR), Deanship of Scientific Research, Taif University, Taif, Saudi Arabia; 5 Pharmacy Practice, AlMaarefa University, Dariyah, Riyadh, Saudi Arabia

**Keywords:** Postpartum depression, Health related quality of life, Pregnancy, Childcare, Mental health, Saudi Arabia, Neonatal care, Mother, Stress

## Abstract

**Background and Objectives:**

The mental and physical functioning of an individual is partly determined by their health-related quality of life (HRQOL), which is a multifaceted component. Women who have recently given birth must have a good quality of life to provide proper care and development for their infant. The purpose of this study was to assess the relationship between postpartum depression (PPD) and HRQOL in Saudi Arabian women and to identify potential risk factors that could influence them.

**Methods:**

This study comprised 253 mothers aged 1–24 weeks postpartum from several health centers in Saudi Arabia, recruited by random purposive sampling. The study’s questionnaire featured three sections: the first section had demographic information; the second and third sections contained the Edinburgh Postnatal Depression Scale (EPDS) scale and the HRQOL scale (SF-12), respectively. Data were analyzed using descriptive statistics, chi square analyses, independent samples t-tests and binary logistic regression analysis using IBM SPSS 25.

**Results:**

Results of current study indicate that 59.68% of the patients exhibited probable post-partum depression symptoms. Participants who were depressed had significantly lower mental component (MCS) and physical component scores than participants who were in good health. When compared to non-smokers, smokers have a 21-fold higher risk of developing depression. Similar to this, mothers who worked had a 3.98 times higher risk of depression, and patients with a history of depression had a 3.6 times higher chance of getting PPD. The probability of developing PPD was also significantly higher in those who lived outside the Riyadh region, had given birth more than twice before this time, and had experienced undesired pregnancies.

**Conclusion:**

Our study demonstrated an inverse correlation between postpartum depression and health-related quality of life scores. Treatment for depression, particularly among mothers, is crucial for improving their quality of life and, as a result, creating a favorable environment for the development of newborn babies.

## Introduction

One of the common mental disorders affecting more than 300 million people globally is depression (American Psychiatric Association, 2013). Depression was ranked fourth by the World Health Organization (WHO) before the turn of the century, but it threatened to jump to second by the end of the century’s first quarter (WHO, 2001). This terrible mental illness has gradually evolved into a disease that threatens hundreds of millions of people around the world and has become a significant contributor to global disability and disease burden (Wang et al., 2021).

Postpartum depression (PPD) is one of the most commonly occurring types of depression among women all over the world (Teissedre & Chabrol, 2004). It is a nonpsychotic depressive illness, defined by the DSM as an episode of significant depression that begins within four weeks of the birth of a child (American Psychiatric Association, 2013). The global prevalence of maternal PPD was reported to be 13% (Gavin et al., 2005). However, there is a lot of variation in the published research on the prevalence of PPD depending on the screening tools and methods utilized, the screening time, and the country in which the study was conducted (Hewitt et al., 2009); for instance, Melo et al. (2012) found only 10.8% of Brazilians had PPD, while Deng et al. (2014) showed 27.37% of Chinese had PPD. In Turkey and Malaysia, PPD prevalence was estimated to be 9.1% (Serhan et al., 2013) and 14.3% (Yusuff et al., 2015), respectively.

In the Middle East, large cross-sectional investigations were conducted. Even though all studies shared comparable socioeconomic conditions, the prevalence rate varied. For example, a study conducted in Sharjah, UAE (Hamdan & Tamim, 2011), noted only a 10% PPD prevalence, whereas a study conducted in Qatar (Burgut et al., 2013) reported a 17.6% PPD prevalence. Furthermore, a survey conducted in Lebanon described only 12.8% of PPD (El-Hachem et al., 2014), whereas an observational study conducted among rural women in Minia, Egypt, revealed 49.5% (Mohammed et al., 2014). PPD prevalence was found to be 17.8% in the Dammam region of Saudi Arabia (Alasoom & Koura, 2014). Another study in Riyadh in the same year found a prevalence of PPD of 33.2% (Alharbi & Abdulghani, 2014). A more recent investigation in Riyadh (Almutairi et al., 2017) confirmed the city’s high frequency of PPD. The discrepancies in healthcare environments, as well as socioeconomic and geographical variables, could explain the variation among various populations.

Postpartum depression results in abnormal behavior of the mother towards her family and newborn (Beck, 1999). Depressed women frequently connect with their infants with minimal responsiveness (Hipwell et al., 2000; Van Doesum et al., 2007) and fail to address their social-emotional requirements (Buist, 1998). Infants of depressed mothers are more likely than those of nondepressed mothers to be mistreated (Drewett et al., 2004), to fail to thrive (Kozyrskyj et al., 2008), to be hospitalized for health problems including asthma (Field, 2010), and to have sleeping problems (Gaffney et al., 2014). Depressed mothers are also more likely to breastfeed infrequently, add cereal to the formula, and begin solid food sooner (Righetti-Veltema, Bousquet & Manzano, 2003). As a result, their babies will attain lower immunological protection and other breast-feeding advantages. PPD has a negative impact on the cognitive development and learning of newborns (Kurstjens & Wolke, 2001). PPD can also lead to suicide and, in rare situations, infanticide if not treated (Cooper et al., 2003).

The specific cause of PPD is unknown. However, researchers have identified several risk factors, including a lack of social support (Brugha et al., 1998), marital conflict (Inandi et al., 2002), a history of depression (Kheirabadi et al., 2009), lack of breastfeeding (Wilson et al., 1996), and recent stressful life events (O’Hara & Swain, 1996). The likelihood of getting PPD has also been linked to unemployment, low education levels, and unintended pregnancies (Ghubash & Abou-Saleh, 1997; Hickey et al., 1997; Patel, Rodrigues & DeSouza, 2002). Validation of reported or additional risk factors for PPD in various cultural settings is required.

Health-related quality of life (HRQOL) is a multidimensional perspective that can be influenced by politics, society, economy, and beliefs and influences an individual’s function in terms of somatic, mental, sociological, and nonphysical aspects of existence (Alhamdan et al., 2017). Women go through a lot of biological, social, and emotional changes during postpartum depression, so they should pay attention to the impact of their quality of life (QOL) at this time. In this course, the QOL helps women to assess their postpartum condition and aids caregivers in improving the health of puerperial women and newborns (Pillitteri, 2010). Previous research in Saudi Arabia has found a link between the severity of PPD and stressful situations as well as spouse support (Al Nasr et al., 2020). The role of PPD in the HRQOL of mothers from the Saudi Arabian region is yet to be explored. Therefore, the purpose of this study was to compare the quality of life of patients with postpartum depression with that of control subjects.

## Materials and Methods

### Study participants

The participants of the study were women (18–50 years) visiting hospitals for a usual postpartum follow-up visit and newborn immunization during the first 1–24 weeks postpartum between October 2020 and April 2021. They were recruited by purposive sampling from Dr. Salman Alhabib Hospital, Takassusi and Olaya, Riyadh; Al Hammadi Hospital, Olaya, Riyadh; Price Sultan Military Hospital, Riyadh; Hospital of Maternity and Children, AlAhsa; and National Guard Hospital, Riyadh. Mothers who gave birth to babies with major congenital anomalies, stillborn, or experienced intrauterine fetal death were excluded, as were those who were having treatment for psychological difficulties.

### Study instrument

There were three sections in the questionnaire. Demographic information was included in the first section, while the Edinburgh Postnatal Depression Scale (EPDS) and the HRQOL scale (SF-12) were included in the second and third sections, respectively.

Demographic variables included socio-demographic characters (age, level of education, employment status, income level, nationality, and location), psychosocial characters (partner support, history of depression, stress life events, social condition, smoking status, and family support), and maternal/child risk factors (mode of delivery, post-partum phase, desire for pregnancy, number of deliveries, breast feeding, use of contraceptives, history of abortion, preterm labor, and neonatal health).

The Arabic version of the Edinburgh Postnatal Depression Scale was adapted from published literature (https://www.healthtranslations.vic.gov.au/resources/edinburgh-postnatal-depression-scale-epds).

The reliability and validity of this Arabic version of the EPDS for screening depression in postpartum women was reported earlier by Ghubash, Abou-Saleh & Daradkeh (1997). The EPDS (Alhamdan et al., 2017) consisted of 10 questions that examine the mother’s emotional experiences during the previous 7 days. Responses were graded on a scale of 0 to 3, with a maximum score of 30, indicating the severity of symptoms. The Cronbach’s alpha of EPDS items in our study was found 0.833. The cut-off points of 12/13 displayed a sensitivity of 84% to 100% and a specificity of 82% to 88%, whereas the cut-off point of 9/10 showed a sensitivity of 68% to 95% and a specificity of 78% to 96% (Murray & Carothers, 1990). Further, a more recent study performed in Saudi Arabia (Almutairi et al., 2017) used 13 or more depression cut-off. Therefore, in the present study, mothers who scored 13 or more on the EPDS were considered probable PPD sufferers, while those who scored 13 were regarded as normal in the current study. For numerical classification of depression, mothers who scored ≥13, 10–12, and 0–9 were categorized as depressed, borderline, and normal, respectively.

The final component of the questionnaire contained the SF-12 (short form survey) items. The Arabic version of SF-12 was thankfully adopted from a study (Alhadhoud et al., 2020) that reported its validity and reliability among the Arabian population. The SF-12 is a suitable substitute for the 36-Item Short Form Health Survey (SF-36) in large surveys of general and targeted populations. The SF-36 provided the raw materials for the entire SF-12.

It includes eight dimensions: physical functioning (PF), role limitations due to physical health problems (RP), bodily pain (BP), general health (GH), vitality (VT), social functioning (SF), role limitations due to emotional problems (RE) and mental health (MH). The subscales PF, RP, BP, and GH forms the physical component summary (PCS-12) scores whereas the subscales VT, SF, RE, and MH forms the mental component summary (MCS-12) scores (Al-Shehri et al., 2008). Each question on the survey has response options with 2 to 6-point scales, as well as a raw score range of 1 to 6. The raw scores are added together and converted to a 0–100 scale (Islam et al., 2017), with a higher score signifying greater health. A web-based scoring application was used to calculate the PCS-12 and MCS-12 scores (www.orthotoolkit.com/sf-12/). Both items of MCS and PCS had good Cronbach’s alpha values of 0.713 and 0.759, respectively.

### Data collection

#### Ethical approval

The research protocol was approved by the institutional review board of AlMaarefa University (201/002/IRB, dated 20/4/2020). All participants agreed to participate in the study by signing a consent form that described the study’s purpose and informed them that doing so entailed giving permission for their replies to be analyzed. They were also assured of the confidentiality.

#### Questionnaire administration

The data collection team consisted of female pharmacy students from AlMaarefa University. This group received adequate training on the method of distribution of questionnaires, its presentation to the intended participants, and gathered feedback. Participants self-administered the study instrument and recorded their responses. However, the data collector carried out the survey through a face-to-face interview for those participants who required assistance in recording their responses.

### Statistical analyses

The data obtained was entered into the IBM SPSS statistical software (version 25). The study sample’s socio-demographic variables were subjected to univariate descriptive analysis and bivariate analysis using the Pearson Chi-square test. Binary logistic regression analysis was used to find the predictors of PPD. All associated variables were compared with HRQOL. The independent samples t-test was utilized to distinguish between the depression and normal groups’ HRQOL mean scores. The chi-square tests were used to determine association between severity of PPD and sociodemographic, psychosocial, maternal, and child risk factors. The different parameters linked to PCS and MCS were compared using multiple linear regression. The level of significance was set at *p* < 0.05 for all inferential procedures.

## Results

### General characteristics of the participants

Table 1 shows that approximately half of the participants were between the ages of 18 and 30 years, with the remaining 50% being older than 30 years. The average age of the participants was 31.45 years (standard deviation: 6.61 years, max 50 years and minimum 50 years). A sizable portion of study participants (69%) were graduates, employed (45%), and had a family income of more than 8,000 SAR (48%). The majority of them were Saudi nationals (91%) with no history of depression (80%). Further, a high percentage of them were married (94%), nonsmokers (91%), and had family support (86%). Additionally, 74% of the participants had normal delivery; 77% wanted to conceive; 74% were breastfeeding their newborns; 78% had no history of abortion; 87% delivered babies at full-term; and 96% of them had healthy newborns.

**Table 1:** General characteristics of the participants.

Characteristics	Variables	Frequency	Percentage
1) Age	18–30 years	129	51
	>30 years	124	49
2) Level of education	Low education*	79	31
	High education**	174	69
3) Employment status	No	140	55
	Yes	113	45
4) Income level	≤8,000 SAR	131	52
	>8,000 SAR	122	48
5) Nationality	Non-Saudi	22	9
	Saudi	231	91
6) Location	Riyadh	144	57
	Out of Riyadh	109	43
7) Partner support	No	244	96
	Yes	9	4
8) History of depression	No	202	80
	Yes	51	20
9) Stressful life events	No	90	36
	Yes	163	64
10) Social condition	Divorced	14	6
	Married	239	94
11) Smoking during pregnancy	No	230	91
	Yes	23	9
12) Family support	Yes	218	86
	No	35	14
13) Mode of delivery	Caesarean	65	26
	Normal	188	74
14) Post-partum phase	Phase 1^#^	107	42
	Phase 2^##^	146	58
15) Desire for Pregnancy	Yes	195	77
	No	58	23
16) Number of deliveries	≤two	131	52
	>two	122	48
17) Breast feeding	No	65	26
	Yes	188	74
18) Contraception use	No	141	56
	Yes	112	44
19) History of abortion	No	198	78
	Yes	55	22
20) Preterm labor	No	221	87
	Yes	32	13
21) Good neonatal health	No	10	4
	Yes	243	96

**Notes:**

# Phase 1: 1–4 weeks postpartum.

## Phase 2: >4 weeks up to 24 weeks postpartum.

* Low education: Secondary, intermediate, and elementary education.

** High education: Graduates and above.

### Sociodemographic characteristic and postpartum depression

Table 2 demonstrates the association between sociodemographic characteristics and the severity of depressive symptoms. Significantly high numbers of working individuals (*p* = 0.003), non-Saudi participants (*p* = 0.011), and those who resided outside Riyadh city (*p* = 0.001) have shown the occurrence of PPD, while the age of the participants, their level of education, and income have not shown any significant impact on the development of postpartum depression.

**Table 2:** Association between sociodemographic characteristic and postpartum depression for chi square analyses.

Socio-demographic characters	Normal *N* (%)^a^	Depression *N* (%)^a^	Total *N*	*p* value^b^
Age				
18–30 years	55 (42.6)	74 (57.4)	129	0.443
>30 years	47 (37.9)	77 (62.1)	124	
Level of education				
Low education^#^	38 (48.1)	41(51.9)	79	0.089
High education^##^	64 (36.8)	110(63.2)	174	
Employment status				
No	68 (48.6)	72 (51.4)	140	0.003**
Yes	34 (30.1)	79 (69.9)	113	
Income level				
≤8,000 SAR	55 (42)	76 (58)	131	0.575
>8,000 SAR	47(38.5)	75(61.5)	122	
Nationality				
Non-Saudi^#^	3 (13.6)	19 (86.4)	22	0.011*
Saudi	99 (42.9)	132 (57.1)	231	
Location				
Riyadh	72 (50)	72 (50)	144	0.001***
Out of Riyadh	30 (27.5)	79 (72.5)	109	

**Notes:**

a Values are given in frequency (percentage).

b *p* value were calculated by Pearson Chi-square test.

# Fisher’s exact test is applied as ‘1 cells’ (25.0%) have expected count less than 5.

# Low education: Secondary, intermediate and elementary education.

## High education: Graduates and above.

* *p* < 0.05.

** *p* < 0.01.

*** *p* < 0.005.

### Psycho-social features and postpartum depression

As exhibited by Table 3, participants who have a history of depression and those who smoke during pregnancy have developed depression in significantly (*p* = 0.001) higher numbers. Although most mothers who did not receive partner support developed depression, this number was low and hence it failed to show a significant impact. Stressful life events, lack of family support, and social conditions have not shown a significant change in the number of depression cases among our participants in this study.

**Table 3:** Association between psycho-social characteristic and postpartum depression for chi square analyses.

Psycho-social characters	Normal *N* (%)^a^	Depression *N* (%)^a^	Total *N*	*p* value^b^
Partner support				
No	100 (41)	144 (59)	244	0.320
Yes	2 (22.2)	7 (77.8)	9	
History of depression				
No	93 (46.0)	109 (54.0)	202	0.001***
Yes	9 (17.6)	42 (82.4)	51	
Stressful life events				
No	43 (47.8)	47 (52.2)	90	0.072
Yes	59(36.2)	104 (63.8)	163	
Social condition				
Divorced^#^	3 (21.4)	11 (78.6)	14	0.169
Married	99 (41.4)	140 (58.6)	239	
Smoking during pregnancy				
No	100 (43.5)	130 (56.5)	230	0.001***
Yes	2 (8.7)	21 (91.3)	23	
Family support				
Yes	93 (42.7)	125 (57.3)	218	0.058
No	9 (25.7)	26 (74.3)	35	

**Notes:**

a Values are given in frequency (percentage).

b *p* value were calculated by Pearson Chi-square test.

# Fisher’s exact test is applied as ‘1 cells’ (25.0%) have expected count less than 5.

*** *p* < 0.005.

### Maternal and child risk factors and postpartum depression

The number of depression cases was high among mothers who did not wish to conceive (*p* = 0.011), delivered more than twice (*p* = 0.004), and those who did not use contraceptives (*p* = 0.009) (Table 4). No significant association was noticed between mode of delivery, postpartum phase, breastfeeding, history of abortion, preterm labor, and regular mental health in the development of postpartum depression.

**Table 4:** Association between maternal and child risk factors and postpartum depression for chi square analyses.

Maternal and child risk factors	Normal *N* (%)^a^	Depression *N* (%)^a^	Total *N*	*p* value^b^
Mode of delivery				
Caesarean	27 (41.5)	38 (58.5)	65	0.816
Normal	75 (39.9%)	113 (60.1%)	188	
Post-partum phase				
Phase 1^#^	50 (46.7)	57 (53.3%)	107	0.075
Phase 2^##^	52 (35.6)	94 (64.4)	146	
Desire for pregnancy				
Yes	87 (44.6)	108 (55.4)	195	0.011*
No	15 (25.9)	43 (74.1)	58	
Number of deliveries				
≤two	64 (48.9)	67(51.1)	131	0.004***
>two	38 (31.1)	84 (68.9)	122	
Breast feeding				
No	20 (30.8)	45 (69.2)	65	0.069
Yes	82 (43.6)	106 (56.4)	188	
Contraception use				
No	35 (31.3)	77 (68.8)	141	0.009**
Yes	67 (47.5)	74 (52.5)	112	
History of abortion				
No	82 (41.4)	116 (58.6)	198	0.499
Yes	20 (36.4)	35 (63.6)	55	
Preterm labor				
No	89 (40.3)	132 (59.7)	221	0.970
Yes	13 (40.6)	19 (59.4)	32	
Good neonatal health				
No	1 (10)	9 (90)	10	0.53
Yes	102 (41.6)	142 (58.4)	243	

**Notes:**

a Values are given in frequency (percentage).

b *p* value were calculated by Pearson Chi-square test.

# Phase 1: 1–4 weeks postpartum.

## Phase 2: >4 weeks up to 24 weeks postpartum.

* *p* < 0.05.

** *p* < 0.01.

*** *p* < 0.005.

### Mental/physical component scores and depression

**Figure 1:**
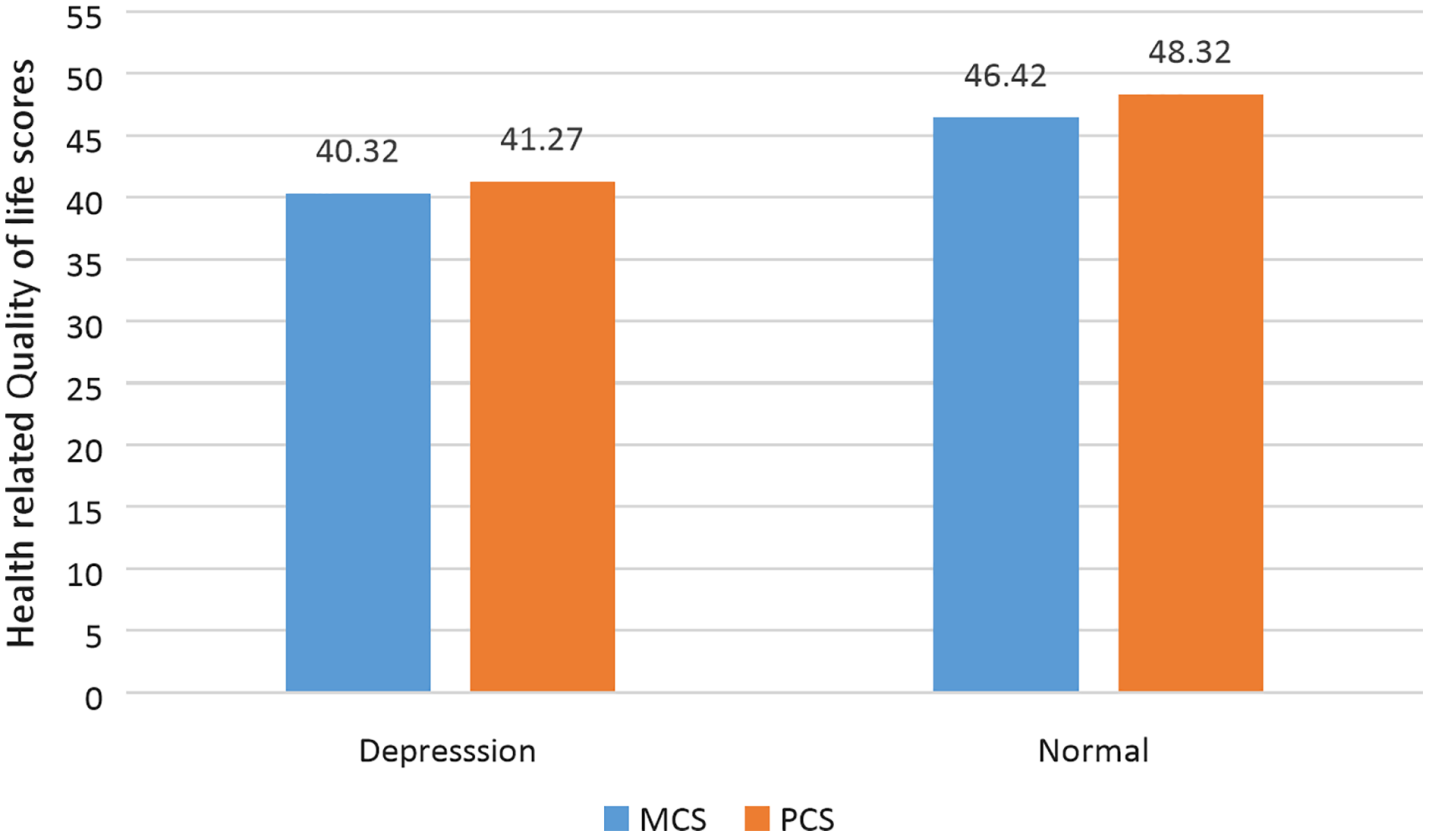
Comparison of mental and physical component scores of HRQOL with depression status.

Depressed participants had significantly lower HRQOL scores (MCS and PCS) than normal participants (Table 5 and Fig. 1). When compared to participants between the ages of 18 and 30 years, participants above the age of 30 years had a significant (*p* < 0.005) decline in both MCS and PCS scores. Further, patients with a history of depression had significantly (*p* < 0.005) reduced PCS and MCS scores compared to those who had no history of depression. Moreover, compared to patients who were not employed, those who were employed had a significantly (*p* < 0.005) increased risk of experiencing a decline in HRQOL.

**Table 5:** Summary of the differences in the means for PCS and MCS in significant variables.

	MCS	*p* value	PCS	*p value*
Depression status				
Yes	40.32 ± 4.32	0.001***	41.27 ± 7.43	0.001***
No	46.42 ± 5.11		48.32 ± 8.21	
Age				
18–30 years	46.04 ± 6.12	0.001***	46.12 ± 7.91	0.001***
>30 years	41.53 ± 6.11		40.40 ± 6.56	
Depression history				
Yes	41.32 ± 8.76	0.002***	41.49 ± 8.21	0.002***
No	45.33 ± 7.10		46.87 ± 6.50	
Employment status				
No	48.11 ± 7.46	0.003***	47.32 ± 5.77	0.003***
Yes	42.98 ± 8.22		43.03 ± 5.32	

**Notes:**

Values are given as mean ± standard error of mean, MCS, Mental component score; PCS, Physical component score.

*** *p* < 0.005.

### Predictors of postpartum depression

Smokers have a 21-fold higher risk of depression than non-smokers. Similarly, employed mothers were 3.98 times more likely to be depressed, and patients with a history of depression were 3.6 times more likely to develop PPD (Table 6). Mothers who had two or more pregnancies before this time had a higher risk of developing PPD. Residents of Riyadh are at a lower risk of PPD than others. In the HRQOL, patients with mental and physical component scores of less than 40 have a significantly higher risk of depression than those with higher scores.

**Table 6:** Predictors of postpartum depression by binary logistic regression analysis.

Independent variables	Wald test	*p value* ^b^	Odds ratio	95% confidence interval odds ratio	
				Lower	Upper
Smoking during pregnancy	7.947	0.005**	21.378	2.543	179.743
Employment status	12.76	0.001***	3.981	1.865	8.495
Depression history	6.619	0.01*	3.652	1.361	9.795
Deliveries range	7.622	0.006**	2.999	1.375	6.541
Location range	7.72	0.005**	2.612	1.327	5.141
MCS	7.838	0.01*	2.644	1.801	4.374
PCS	7.592	0.01*	2.794	1.881	4.656
History of stress	3.775	0.052	2.108	0.993	4.472
Postpartum phase	3.51	0.061	1.894	0.971	3.695
Age range	2.053	0.152	1.779	0.809	3.914
Marital status	0.182	0.67	1.482	0.243	9.041
Education range	0.074	0.785	1.108	0.529	2.321
History of abortion	0.02	0.888	1.065	0.442	2.569
Income range	0.019	0.891	1.051	0.518	2.133
Mode of delivery	0.001	0.974	1.012	0.482	2.128
Family support	0.155	0.694	0.806	0.275	2.359
Breast feeding	1.315	0.251	0.628	0.284	1.39
Preterm delivery	1.191	0.275	0.546	0.184	1.618
Contraceptive Use	5.117	0.024	0.444	0.22	0.897
Status of stillbirth	1.015	0.314	0.431	0.084	2.214
Desire for pregnancy	4.808	0.028*	0.389	0.167	0.905
Violence from spouse	0.873	0.35	0.336	0.034	3.314
Nationality	2.334	0.127	0.321	0.075	1.379
Good neonatal health	2.04	0.153	0.142	0.01	2.07

**Notes:**

Independent variables are arranged in the decreasing order of their association with postpartum depression.

b *p* value were calculated by Pearson Chi-square test.

* *p* < 0.05.

** *p* < 0.01.

*** *p* < 0.005.

## Discussion

The purpose of this cross-sectional study was to determine the prevalence of postpartum depression (PPD), its predictors, and the impact of PPD on the health-related quality of life among the Saudi Arabian female population. The results suggested a high prevalence of PPD among study participants. PPD is more likely to occur in mothers who are employed, smoke regularly, have a prior history of depression, have given birth frequently, and have had unplanned pregnancies. The study’s findings highlighted the significance of taking appropriate action to fight depression and encouraged mothers to lead healthy lifestyles in order to support the healthy growth and development of their newborns.

The World Health Organization estimates that 20–40% of people in developing nations suffer from PPD. This study’s PPD prevalence was close to 60%, which is greater than that of a prior study carried out in the Riyadh region (Ajinkya, Jadhav & Srivastava, 2013). COVID-19 has an undeniable effect on the overall population. Asdaq et al. (2020) found a 44% prevalence of depression in Riyadh’s general female population during the early stages of COVID-19. Because this study was conducted during COVID-19, it is likely that the pandemic exacerbated the burden of depression among Saudi Arabian mothers.

Our study documented an almost four-times higher risk of PPD in working women than in those who are not working. Our findings are consistent with a previous study that found that one in every five working women in Canada had either been diagnosed with or fulfilled the diagnostic criteria for major depression (Depression & Anxiety, 2004). Perceived negative work interactions and job insecurity are all key factors that are thought to increase depression risk (Seto, Morimoto & Maruyama, 2004). Mothers who give birth might struggle to control this circumstance and might display higher levels of PPD.

Although the number of mothers who confess to smoking during pregnancy is lower, there is a significantly greater number of depression rates found among smokers. Smokers are up to 21 times more likely than non-smokers to experience postpartum depression. This is consistent with an earlier meta-analysis that emphasized that smokers are more likely to report depressive symptoms than non-smokers (Zhu & Valbø, 2002). To improve the quality of life for both mothers and newborns, harmful lifestyle choices such as smoking must be avoided by mothers.

In our study, patients who had a history of depression had a roughly threefold higher risk of developing depression. A study done on more than 700,000 Swedish mothers between 1997 and 2008 showed a 20-fold higher risk of PPD than those without a history of depression (Silverman et al., 2017). Therefore, it is crucial to offer additional care following delivery when dealing with mothers who have a history of depression.

Another interesting finding of this study is the lower rate of depression among residents of Riyadh than those who reside outside Riyadh. The capital of Saudi Arabia, Riyadh, is a modern city that provides more mental health services than other cities. Mothers who live outside of Riyadh suffer a 2.6-fold increased risk of depression. In Saudi Arabia, as in the rest of the world, urbanization has been and will continue to be the predominant trend in population expansion. More than half of the world’s population now resides in urban areas, and this trend is just continuing to grow. Generally, it is believed that urbanization is detrimental to mental health. However, there are a number of recent reports that support our findings that urbanization has an association with a diminished depression rate. A study done in China exhibited that depression prevalence decreases with an increase in urbanization to a significant level (He, Song & Jian, 2020). A more recent study (Stier et al., 2021) done in the United States provided further evidence of a decreasing depression rate among residents of larger cities. Urbanization helps in improving the quality of life by supporting systems such as better access to health care, employment opportunities, education, and other services (Forouzandeh, Delaram & Deris, 2003).

The significance of health-related quality of life (QOL) in relation to maternal health and pregnancy outcomes has been shown in earlier research (Deng et al., 2014). Our study demonstrated that mothers who were depressed had a lower quality of life (QOL) than mothers who were not depressed. The findings of this study concur with those of Forouzandeh, Delaram & Deris (2003), who hypothesized that mothers with depression would experience decreased mental and physical health. Further research (El-Hachem et al., 2014) linked the poor mental and physical health of depressed mothers to reduced QOL. A study (De Tychey et al., 2008) on 181 PPD mothers in France found impaired physical and mental components of SF-36 subscales. Poor personal care, household maintenance, employment, and social engagement were observed among mothers with PPD in a cross-sectional study conducted in the United States (Posmontier, 2008) compared to mothers without PPD in a cross-sectional study conducted in the United States (Posmontier, 2008). Additionally, Zubaran et al. (2010) in Southern Brazil investigated 101 mothers between the second and twelfth weeks postpartum and discovered that there was a significant inverse relationship between PPD and health status.

Our study’s results indicate that PPD has a significant negative impact on all aspects of life quality, including physical and mental health. In any case, depression during pregnancy may distort the estimated difference in QOL ratings. We are aware that the lack of information on prenatal depression and prenatal QOL places constraints on our investigation. This study’s use of a universal QOL measurement tool was another limitation. However, it was a prospective study utilizing an instrument specifically designed for PPD (the EPDS). Further, because there are many expatriates in Saudi Arabia, the results of this study cannot be generalized to all groups of Saudi Arabians as there were less than 10% of non-Saudi participants included in this study. This may have been avoided by increasing the sample size and diversifying the sampling sites across the country’s health centers or various regions. In addition, data regarding the individuals’ comorbid disorders other than their history of depression was not gathered. Numerous comorbidities have the potential to either directly or indirectly raise the prevalence of depression. However, the individuals’ average age was close to thirty, which may have reduced the likelihood that they had any serious comorbidities. Finally, the Edinburgh Postnatal Depression Scale (EPDS) is only based on participants’ recall of assertions they made about their feelings as well as their capacity to carry out specific tasks over the previous seven days. The total incidence is simply a trend, and the true frequency of PPD may vary, as the EPDS is only a tool for detecting depression symptoms. Nevertheless, our findings emphasized that PPD screening be incorporated into standard postnatal treatment since research on postpartum QOL and related issues, such as PPD, can be helpful for preventative programs and the care of postpartum mothers.

## Conclusion

Postpartum depression was seen in a large number of the study’s participants. Women who smoke have a higher risk of depression, which is subsequently followed by working mothers and women who have previously had depression and are expecting their third or subsequent child. The health-related quality of life score was found to be negatively correlated with depression. Screening high-risk mothers for PPD during and after pregnancy is necessary for the early identification and prevention of family psychological damage. Treatment for depression, particularly for mothers, is essential to enhance their quality of life and, as a result, creating a favorable environment for the growth of newborn children.

## Supplemental Information

10.7717/peerj.14240/supp-1 Supplemental Information 1 Raw data.

10.7717/peerj.14240/supp-2 Supplemental Information 2 Arabic version of EPDS.

## Additional Information and Declarations

### Data Availability

The following information was supplied regarding data availability: The raw data are available in the Supplemental File.
