# Supplementary material for: Postpartum depression and health-related quality of life: a Saudi Arabian perspective
Source: PeerJ. 2022 Oct 14;10:e14240. doi: 10.7717/peerj.14240 (PMC9575671; doi:10.7717/peerj.14240)
Supplement: Supplemental Information 2 [file peerj-10-14240-s002.pdf]

NSW GOVERNMENT **Health**

| FAMILY NAME | MRN |
|---|---|
| GIVEN NAME | MALE FEMALE |
| D.O.B: / / | M.O. |
| ADDRESS: | |
| | |
| LOCATION: | |
| COMPLETE ALL DETAILS OR AFFIX PATIENT LABEL HERE | |

**Facility:**

## The Edinburgh Postnatal Depression Scale

Cox JL, Holden JM Sagovsky R (1987) Detection of postnatal depression: development of the 10-item Edinburgh postnatal depression scale. Brit J Psychiatry150 782-86. Reproduced with permission

ARABIC

**بما أنك أنجبت طفلاً مؤخرًا، نود أن نعرف شعورك الآن. يرجى وضع علامة في خانة الإجابة التي تعبر بشكل أفضل عما شعرت به في الأيام السبعة الماضية، وليس فقط عما تشعرين به اليوم. إليك هذا المثال للتوضيح.**

**شعرت بأنني سعيدة**

- ☐ نعم، في جميع الأوقات
- ☑ نعم، في معظم الأوقات
- ☐ كلا، ليس كثيراً
- ☐ كلا، أبداً

وهذا يعني: "شعرت بأنني سعيدة معظم الوقت خلال الأسبوع الماضي". نرجو منك أن تكملي الأسئلة الأخرى بالطريقة ذاتها.

**.1 استطعت الشعور بالفرح والسعادة:**

- ☐ بالمقدار نفسه الذي استطعته من قبل
- ☐ ليس تماماً بالمقدار نفسه الآن
- ☐ ليس بالمقدار نفسه الآن
- ☐ كلا مطلقاً

**.2 تطلعت إلى الأمور بتشوّق:**

- ☐ بالمقدار نفسه مثل أي وقت مضى
- ☐ أقل نوعاً ما مما اعتدته
- ☐ أقل بكثير مما اعتدته
- ☐ نادراً

**.3 لمت نفسي بدون لزوم عندما سارت الأمور على غير ما يرام:**

- ☐ نعم، في معظم الأحيان
- ☐ نعم، في بعض الأحيان
- ☐ ليس في أحوال كثيرة
- ☐ كلا، أبداً

**.4 كنت قلقة ومشغولة البال بدون سبب وجيه:**

- ☐ كلا، أبداً
- ☐ نادراً
- ☐ نعم، في بعض الأحيان
- ☐ نعم، في كثير من الأحيان

**.5 شعرت بالخوف والذعر بدون سبب وجيه:**

- ☐ نعم، كثيراً في كثير من الأحيان
- ☐ نعم، في بعض الأحيان
- ☐ كلا، ليس كثيراً
- ☐ كلا، مطلقاً

**.6 تراكمت الأعمال عليّ فلم أستطع القيام بها كلها:**

- ☐ نعم، في معظم الأحيان لم أستطع القيام بها أبداً
- ☐ نعم، في بعض الأحيان لم أستطع القيام بها كالمعتاد
- ☐ كلا، لقد استطعت القيام بها في بعض الأحيان
- ☐ كلا، لقد استطعت القيام بها كالمعتاد

**.7 كنت تعيسة لدرجة أنني واجهت صعوبة في النوم:**

- ☐ نعم، في معظم الأحيان
- ☐ نعم، في بعض الأحيان
- ☐ كلا، ليس كثيراً
- ☐ كلا، أبداً

**.8 شعرت بأنني تعيسة وبائسة:**

- ☐ نعم، في معظم الأحيان
- ☐ نعم، في أحيان كثيرة
- ☐ ليس كثيراً
- ☐ كلا، مطلقاً

**.9 شعرت بالتعاسة لدرجة البكاء:**

- ☐ نعم، في معظم الأحيان
- ☐ نعم، في كثير من الأحيان
- ☐ من وقت لآخر فقط
- ☐ كلا، مطلقاً

**.10 خطرت لي فكرة إلحاق الأذى بنفسي:**

- ☐ نعم، في أحيان كثيرة
- ☐ أحياناً
- ☐ نادراً
- ☐ كلا، مطلقاً

**Date Completed:** ______________________ **Total Score:**__________/ 30 **Total Score for Question 10:** ____/ 3

Holes Punched as per AS2828.1: 2012
BINDING MARGIN - NO WRITING
